# Antimicrobial Stewardship in Pediatric Emergency Medicine: A Narrative Exploration of Antibiotic Overprescribing, Stewardship Interventions, and Performance Metrics

**DOI:** 10.3390/children11030276

**Published:** 2024-02-23

**Authors:** Kevin Meesters, Danilo Buonsenso

**Affiliations:** 1 Department of Pediatrics, Faculty of Medicine, University of British Columbia, Vancouver, BC V6H 3V4, Canada; 2 Vaccine Evaluation Center, BC Children’s Hospital Research Institute, Vancouver, BC V5Z 4H4, Canada; 3 Department of Woman and Child Health and Public Health, Fondazione Policlinico Universitario Agostino Gemelli IRCCS, Universita Cattolica del Sacro Cuore, 00168 Rome, Italy; danilo.buonsenso@policlinicogemelli.it; 4 Centro di Salute Globale, Universita Cattolica del Sacro Cuore, 00168 Rome, Italy

**Keywords:** antimicrobial stewardship, pediatric emergency medicine, biomarkers, rapid antigen detection tests, guidelines, education, feedback, defined daily dose, days of therapy

## Abstract

Antibiotic overprescribing is prevalent in pediatric emergency medicine, influenced by clinician–caregiver dynamics and diagnostic uncertainties, and poses substantial risks such as increasing antibacterial resistance, adverse drug reactions, and increased healthcare expenditures. While antimicrobial stewardship programs have proven effective in optimizing antibiotic use within inpatient healthcare settings, their implementation in pediatric emergency medicine presents specific challenges. Existing biomarkers like white blood cell count, C-reactive protein, procalcitonin, and presepsin have limitations in their ability to distinguish (serious) bacterial infections from other etiologies of fever. Furthermore, rapid antigen detection tests and guidelines aimed at guiding antibiotic prescriptions for children have not consistently reduced unnecessary antibiotic use. To improve antibiotic prescribing practices, potential strategies include the utilization of decision support tools, audit and feedback, establishing follow-up procedures, implementing safety netting systems, and delivering comprehensive training and supervision. Notably, host genome signatures have also gained attention for their potential to facilitate rapid and precise diagnoses of inflammatory syndromes. Standardized metrics are crucial for evaluating antimicrobial use within pediatric healthcare settings, enabling the establishment of benchmarks for assessing antibiotic utilization, quality enhancement initiatives, and research endeavors.

## 1. Introduction

The frequency of children seeking medical care at emergency departments (EDs) has consistently increased on a global scale during the last decades [1,2,3,4,5], with fever, cough, and upper respiratory tract infections (RTI) being the most common reasons for ED attendance. There are notable variations among emergency medical services, both at the local and international levels, in terms of pediatric patient volume, referral and hospitalization rates, and the specific medical specialties overseeing pediatric care [6,7]. These discrepancies pose challenges when attempting to make international comparisons. However, the overall factors driving the increase in ED use encompass the strain on primary healthcare facilities and the practical advantages of EDs, such as their constant availability and direct access to diagnostic investigations [8,9,10]. A considerable proportion of children receiving antibiotic prescriptions in EDs is evident, with reported rates ranging from 19% in Switzerland to 64% in Turkey [11]. A more recent report indicated that 46.3% of all children who attended EDs across various European countries and manifested a viral phenotype received antibiotic prescriptions, frequently involving second-line antibiotics [12]. The utilization of antibiotics invariably contributes to the development of antibacterial resistance (ABR), and prescribing antibiotics within an ED leads to extended hospital stays and increased healthcare expenditures [13,14,15]. Antimicrobial stewardship (AMS) programs have been gradually established across various inpatient healthcare settings, leading to decreases in antimicrobial use, costs, and prescription errors, all without compromising patient safety [16,17]. Yet, while the majority of antibiotics are prescribed to children treated as outpatients [18,19], common AMS techniques such as audits and feedback, antibiotic time-outs, and formulary restrictions encounter challenges when applied within ED settings [17]. In this narrative review, we outline the diagnostic uncertainties that surround antibiotic prescription to children within ED settings. Subsequently, we present a comprehensive overview of potential AMS techniques applicable to pediatric emergency medicine (PEM), along with the assessment tools to evaluate the impact of these interventions.

## 2. Factors Affecting Antibiotic Prescribing in PEM

The decision to prescribe antibiotics to a pediatric patient, whether warranted by clinical indications or not, involves a complex interplay among the clinician, the patient, and their respective caregivers. In the context of EDs, the prevailing diagnostic approach tends to favor speed and intuition over deliberate and comprehensive evaluations. Qualitative investigations into this domain have brought to light a spectrum of cognitive biases that exert notable influence over decisions pertaining to antibiotic therapy; these are outlined in Table 1.

Clinicians working in the ED often perceive a demand for antibiotics from the caregiver [20]. Yet, studies examining the drivers behind ED attendance commonly reveal that caregivers primarily seek explanations for their child’s fever, reassurance, and guidance on symptom management [21,22,23]. Childhood infections, particularly those characterized by fever and respiratory complaints, induce discomfort in children and anxiety in caregivers, compelling them to seek urgent medical attention. External pressures, such as the desire to be perceived as responsible caregivers by family members and childcare providers, may further intensify this perceived urgency [4,24,25].

While many caregivers are cognizant of the issue of antimicrobial resistance, their knowledge of the judicious use of antibiotics remains limited [26,27,28]. Exemplarily, the difference between bacterial and viral diseases is poorly understood [21,29]. In a cross-sectional study conducted in Greece, 86% of parents acknowledged that the majority of infections displaying common cold or flu symptoms were attributed to viruses, yet 26.9% held the belief that antibiotics could potentially prevent complications [30]. Caregivers report confusing messages about the indications for antibiotics. For instance, in Finland, where antibiotics are recommended for children with otitis media, caregivers are more inclined to anticipate antibiotics compared to the Netherlands, where a more conservative approach is advised [31]. Moreover, caregivers whose children were previously prescribed were more likely to believe that antibiotics are necessary in subsequent encounters [32]. Additionally, requests for antibiotics may stem from fears of protracted or complicated infectious illnesses [33,34]. Caregivers place great emphasis on fostering open communication with clinicians, valuing a relationship built on trust and characterized by the provision of comprehensive information [34,35]. Within this context, it is noteworthy that characterizing an illness as viral can elicit specific perceptions and reactions among caregivers, potentially minimizing the significance of their child’s symptoms and leading to a perceived downgrading of the condition [22,36].

Clinicians in various settings acknowledge that antibiotics are frequently inappropriately prescribed, particularly during out-of-hours service [37]. However, healthcare providers often do not identify themselves as overprescribers [38]. Factors affecting antibiotic prescription in RTI include the duration of symptoms, preferences of caregivers, and self-management practices, as well as the treatment approaches employed by their colleagues. Furthermore, injudicious use of resources, in particular rapid antigen detection tests, increased antibiotic prescriptions in multiple settings [39,40,41]. Some clinicians admit to prescribing antibiotics sooner to circumvent prolonged discussions with caregivers during time-pressured consultations or suggest they are driven by a fear of losing patients if antibiotics are not prescribed [21].

Remarkably, in a qualitative interview-based study involving British general practitioners addressing pediatric RTI, clinicians tend to refrain from categorizing themselves as overprescribers, suggesting a perceived lack of necessity to alter their prescribing practices [42]. Furthermore, a qualitative observational study conducted in the UK, employing video recordings of consultations involving children with RTI in primary care settings, reveals that antibiotic prescriptions were not primarily motivated by requests of caregivers but rather by prescribers’ concerns regarding the potential presence of bacterial infections when atypical symptoms were observed [43]. Similarly, an interview-based study in the USA involving primary care pediatricians found that while these healthcare providers acknowledged antibiotic overprescription, they did not perceive themselves as significant contributors to the issue. Moreover, there was a prevalent distrust of audit data, with pressures from caregivers being cited as a factor affecting antibiotic prescriptions [44]. In a survey of French general practitioners, 63% of respondents concurred that upper RTI was overly treated with antibiotics, while 27.1% believed that upper RTI could potentially lead to complications that should be avoided by prescribing antibiotics [45].

Conversely, caregivers’ satisfaction does not appear to be contingent on the prescription of antibiotics for their children’s ailments. In fact, the majority of caregivers expressed strong disagreement with the notion that they would seek an alternative clinician’s care if antibiotics were not prescribed to address their child’s RTI [21]. Instead, a significant portion of caregivers (66%) expressed a preference for receiving a diagnosis, management advice, or reassurance for their febrile child, while only a small minority (8%) explicitly expected antibiotics. It is noteworthy that a considerable proportion (72%) of caregivers displayed an awareness that cold and flu illnesses are primarily caused by viruses, rendering antibiotics ineffective against such infections. Similar findings were obtained in a Dutch interview study [38].

In essence, these findings underscore the importance of effective communication in the clinician–caregiver relationship. While caregivers value trust and comprehensive information, their satisfaction and expectations regarding antibiotic prescriptions for their children appear to be more closely linked to the need for diagnosis, guidance, and reassurance rather than an inherent demand for antibiotics.

**Table 1:** Cognitive biases that affect antibiotic decision making.

Bias	Definition	Example
Anticipated regret	Cognitive processes where individuals anticipate the potential negative consequences or regrets associated with a decision and take proactive steps to mitigate those regrets [46].	Prescribing amoxicillin for an infant with bronchiolitis at the onset of symptoms ‘just in case’ as a precautionary measure against a potential secondary bacterial infection, with the intent of preventing subsequent deterioration.
Anchoring and adjustment	The tendency to rely on the initial piece of information encountered (the anchor) and then adjust subsequent decisions based on the initial anchor [47].	Ordering a rapid streptococcal antigen test in a toddler with fever, nasal congestion, pharyngitis, and cough with the primary objective of initiating antibiotic therapy in the event of a positive test result.
Confirmation bias	The inclination to give precedence to information that aligns with an initial or desired hypothesis while overlooking evidence in other directions [48].	Perceiving the presence of white plaques on the tonsils as indicative of bacterial tonsillitis, while that could also align with viral infections.
Availability bias	Propensity to overestimate the probability on events that are easily recalled [49].	Experiencing a high degree of confidence in diagnosing a pediatric patient exhibiting symptoms of fevers, rigors, and myalgias with meningococcal septicemia, when, in reality, the likelihood of an influenza infection in significantly more substantial.
Representativeness heuristic	Making decisions based on how similar something is to a prototype from a category [50].	Diagnosing a child with unexplained fever and pyuria with pyelonephritis without ordering a urine culture. However, both symptoms could be well in keeping with hyperinflammatory conditions (e.g., Kawasaki disease).
Commission and omission bias	The tendency towards taking action rather than remaining inactive (commission) or inaction instead of action (omission) [51].	Commission: Prescribing antibiotics for an uncomplicated RTI because they want to take action and address the concerns.Omission: Not prescribing antibiotics to an infant with acute otitis media out of concern for potential adverse effects of antibiotics, while this child could benefit from antibiotic therapy.
Hyperbolic discounting/present bias	Propensity to prioritize minor immediate gains over greater, distant benefits or diminished adversities [52].	During a busy shift, a clinician assesses a child with 4 days of fever, coryza, and cough. Instead of an assessment with CRP and chest X-ray, the clinician decides to prescribe antibiotics straightaway, even though they are aware of the potential risks of overuse and the importance of an accurate diagnosis.
Optimism bias	Tendency to perceive one’s own task as unique, leading to the belief that it will remain unaffected by setbacks [51].	Despite the patient’s symptoms and medical history suggesting a probably viral cause for the illness, a clinician believes that it is a bacterial infection that will respond well to antibiotics.
Negativity bias	The inclination to assign greater significance to negative experiences or information compared to neutral or positive ones [51].	During the assessment of a child with fever, the clinician is particularly focused on the potential negative consequences of antibiotic use, such as antibiotic resistance and adverse effects. They are strongly inclined to avoid prescribing antibiotics at all costs due to their heightened concern about these negative outcomes.
Diagnostic momentum	Persisting with a clinical course of action initiated by prior clinicians without adequately factoring in the available information and adjusting the approach when necessary, especially if the plan was initiated by a more senior clinician [53].	An infant with typical symptoms of bronchiolitis presents at the ED. He is not needing oxygen and he is otherwise stable. A chest X-ray, requested as he was referred by his primary care pediatrician to rule out pneumonia, reveals nonspecific bronchial wall thickenings. The initial hypothesis of pneumonia by the primary care pediatrician affected subsequent decisions in the ED.
IKEA effect	The inclination to attribute greater value and satisfaction to a hypothesis or product that they personally crafted [52].	A child presents with one day of fever, rhinorrhea, and irritability. The pediatrician assumes this is acute otitis media, and otoscopy reveals bilateral red tympanic membranes with cones of light present. The resident mentions that these are nonspecific findings and could be explained by both upper RTI and crying. Nevertheless, the pediatrician elects to prescribe antibiotics for acute otitis media.

## 3. Defining AMS in a Global Context

The realization that the development of ABR is not keeping pace with the invention of new antibiotics to treat resistant organisms has prompted the introduction of the term AMS [54]. The variability in AMS definitions contributes to differences in the availability and content of AMS activities worldwide, making comparisons between different activities challenging. However, a universally accepted definition for this concept is currently lacking. While the overarching goals of AMS typically revolve around improving antibiotic therapies for patients, minimizing ABR, and mitigating adverse effects, different healthcare centers have provided diverse operationalizations of AMS activities. Some centers have established multidisciplinary teams comprising clinicians, pharmacists, microbiologists, and infection prevention and control specialists among others. These teams focus on critical aspects such as selecting the right drug at an appropriate dose, determining the optimal duration of therapy, and advocating for de-escalation to pathogen-targeted therapy whenever possible.

The Infectious Diseases Society of America (IDSA) defines AMS as “coordinated interventions designed to improve and measure the appropriate use of antimicrobial agents by promoting the selection of the optimal antimicrobial drug regimen, including dosing, duration of therapy, and route of administration” [55]. In parallel, the ESCMID (European Society of Clinical Microbiology and Infectious Diseases) Study Group for Antimicrobial Stewardship (ESGAP) proposed a broader definition, describing AMS as “a coherent set of actions which promote using antimicrobials responsibly” [56]. This expansive definition underscores that the responsibility for AMS is not limited solely to prescribers and other direct healthcare staff members, such as nurses. It also involves active participation from patients, who play a role in using antibiotics responsibly, as well as contributions from hospital governance (e.g., through funding AMS activities), agricultural workers (e.g., by not using antibiotics as growth promoters in animals), and pharmaceutical companies (e.g., by ensuring the supply of narrow spectrum antibiotics).

## 4. From Biomarkers to Safety Nets: An Exploration of Potential AMS Interventions in PEM

### 4.1. Biomarkers

Serious bacterial infections (SBI) constitute significant contributors to childhood mortality and morbidity worldwide [57]. As clinical signs and symptoms alone fail to distinguish SBI from other causes of fever, there is a pressing need in PEM research to identify biomarkers for SBI recognition. The absence of accurate gold standard tests for some SBIs, such as pneumonia and sepsis, presents a primary hurdle in the validation of these biomarkers [58].

To date, conventional laboratory markers, including white blood cell count (WBC), C-reactive protein (CRP), and urinalyses, encompass the most readily accessible biomarkers, although with imperfect accuracy [59]. For instance, while urinalyses reliably detect urinary tract infection (UTI) in young infants [60], they lack predictive value for concurrent meningitis in this population [61].

CRP, an acute-phase reactant primarily synthesized in the liver and responsive to inflammatory cytokines, promotes bacterial phagocytosis. Its physiological levels have been widely studied, even within the initial hours of life [62]. Compared to white blood cell count (WBC), absolute neutrophil count (ANC), and neutrophil-to-lymphocyte ratio (NLR), CRP exhibits superior discriminatory performance for SBI in the context of young infants presenting with fever in the ED, as evidenced by an area under the receiver operator curve (ROC) of 0.815 (95% confidence interval (CI) 0.748 to 0.883) [63]. It is crucial to emphasize that none of these individual biomarkers achieve perfection, aligning with findings from prior studies. Furthermore, a significant limitation of CRP is its delayed elevation, typically manifesting 4 to 6 h post-symptom onset and peaking 24 to 48 h later. Notably, in the study by Chang et al., 13 infants (24.1%) with SBI and 2 infants (33.3%) with invasive bacterial infections (IBI) were erroneously classified. This includes a 77-day-old infant with Group B Streptococcus (GBS) bacteremia and GBS meningitis, who initially presented with a CRP level of only 1.8 mg/L [63]. Senn et al. conducted a systematic literature review assessing the impact of CRP point of care on antibiotic prescription for RTI in primary care. Thirteen studies of moderate to high quality and involving 9844 participants met the inclusion criteria [64]. The analysis revealed that point-of-care CRP significantly reduced immediate antibiotic prescription compared to usual care (relative risk 0.79, 95% CI 0.70 to 0.90, *p* = 0.0003), though no significant effect was observed during the 28-day follow-up period. Conversely, a previous Cochrane analysis that evaluated the role of CRP in reducing antibiotic prescriptions found only a marginal potential reduction in antibiotic prescriptions, potentially offset by an increased risk of hospitalization [65].

Procalcitonin (PCT) has emerged as a potential marker for bacterial infections; it is primarily produced in the liver in response to cytokines (including IL-6, TNF-α, and IL-1β). Importantly, PCT production is reduced under the influence of interferon-γ, which is typically associated with viral infections and could therefore be a more specific biomarker for SBI [66]. In adults, PCT has shown promise in reducing antibiotic therapy duration without apparent harm [67]. A meta-analysis of 7260 children without an apparent fever source examined the diagnostic accuracy of PCT. At a threshold of 0.5 ng/mL, PCT demonstrated a sensitivity and specificity of 82% and 86% for IBI, and at 2 ng/mL, these values were 61% and 94% [68]. However, PCT performance was less robust in detecting SBI, with 55% sensitivity and 85% specificity at the lower threshold and 30% sensitivity and 95% specificity at the higher threshold. Therefore, PCT may be more valuable in identifying IBI but may miss less severe infections. However, in areas with higher SBI incidence and delayed ED presentations, PCT showed better diagnostic utility [69]. Yet, recent studies have raised questions about the effectiveness of PCT-guided antibiotic therapy in febrile children. An Italian study found PCT to be less accurate in diagnosing SBI compared to CRP [70]. A randomized controlled trial involving children with fever at a pediatric ED that compared decision making based on PCT versus conventional care showed no benefits in terms of reducing antibiotic prescriptions [71], as also suggested by a systematic review [72]. The effect of PCT on antibiotic decision making may be more pronounced in lower RTI, where viral etiologies prevail but antibiotics are often prescribed. A systematic review with meta-analysis found PCT outperformed WBC and erythrocyte sedimentation rate (ESR) in identifying pneumonia but exhibited suboptimal sensitivity [59]. Moreover, several studies investigated the role of PCT in supporting antibiotic duration in children. Two trials and one retrospective study suggested that low PCT concentrations, particularly in well-appearing children, effectively reduced antibiotic use without increasing adverse outcomes [73,74,75]. PCT also demonstrated utility as a marker of disease severity, with significantly higher levels in children with severe community-acquired pneumonia (CAP) [76,77], potentially aiding decisions on antibiotic duration rather than determining which children should initiate antibiotic therapy. Furthermore, in bronchiolitis cases where bacterial superinfections are rare yet antibiotics are often overused, PCT emerged as a potential tool for reducing antibiotic prescriptions [78]. Of note, false positive results are also well described in children with viral infections, especially adenovirus infections, that are associated with a still relatively unexplained spike in inflammatory markers [79].

Presepsin, the N-terminal fragment of the CD14 protein [80], is a cell surface glycoprotein expressed in macrophages, monocytes, dendritic cells, and neutrophils. It acts as a receptor for lipopolysaccharides that triggers the release of proinflammatory cytokines and activates a systematic inflammatory response [81]. Following infection onset, presepsin levels rise within 2 h, reaching their peak at 3 h, and can be measured with point-of-care tests after 3 h [82]. The role of presepsin as a predictor of bacterial infections has gained interest in pediatrics, although it has mainly been studied in adults and to a lesser extent in newborns [83,84]. A systematic review and meta-analysis including 308 pediatric patients (aged 1 to 18 years) reported a sensitivity of 94% (95% CI 74–99%) and specificity of 71% (95% CI 35–92%) [85]. The pooled diagnostic odds ratio, positive likelihood ratio (LR), and negative LR for presepsin were 32.87 (95% CI 2.12–510.09), 3.24 (95% CI 1.14–12.38), and 0.08 (95% CI 0.01–0.74), respectively. Notably, the sensitivity (94%) surpassed that of CRP (51%) and PCT (76%), while the overall specificity (71%) was lower than CRP (81%) and comparable to PCT (76%).

In the endeavor to differentiate bacterial infections from alternative causes of fever, there has been a notable shift in research focus from relying solely on individual biomarkers to embracing multifaceted approaches. Carlton et al. conducted a systematic review on the combination of different biomarkers, in which studies in both adults and children were included. Sensitivities of 61–100% and specificities of 18–96% were found for bacterial etiologies and sensitivities of 59–97% and specificities from 74 to 100% were found for viral etiologies [86]. 

Furthermore, further advancement in this context is the utilization of host RNA signatures, a novel tool designed to precisely discriminate between various inflammatory responses by scrutinizing the transcriptional biosignatures of RNA within host leukocytes [87]. Among the diverse biosignatures investigated, certain molecules have garnered significant attention, including plasmalemma vesicle-associated protein 1 (PV-1), intercellular adhesion molecule-1 (ICAM-1), and phospholipase A2 (PLA2) [70]. ICAM-1, in particular, exhibits promise due to its pivotal role in cell adhesion and immune responses. As a member of the cell surface immunoglobulin superfamily of adhesion receptors, ICAM-1 contributes to critical functions, such as lymphocyte-mediated adhesion, cytotoxic T cell activity, and antigen presentation, and acts as a ligand for the macrophage-associated complex (MAC-1) [88]. Emerging evidence also suggests its potential utility in the early diagnosis of sepsis in newborns and infants, with potential links to the severity of the condition [89,90]. Concurrently, different transcriptomic signatures have been analyzed in diverse cohorts of patients. Although these findings have not yet been seamlessly integrated into routine clinical practice, the development of pocket-sized devices designed for point-of-care implementation has commenced [91]. This progressive body of evidence increasingly supports the notion that transcript host RNA signatures hold the potential to substantially enhance the characterization of a wide spectrum of febrile illnesses in children, encompassing viral, bacterial, and inflammatory etiologies. These signatures can complement traditional diagnostic methods, aiding clinicians in determining the appropriate timing and necessity of commencing antibiotic therapy [92].

### 4.2. Rapid Antigen Detection Tests

Children with RTI are often prescribed antibiotics, even though it is widely established that viruses represent the predominant cause. Therefore, point-of-care rapid viral testing for multiple respiratory viruses, with results obtainable within 40–70 min, could reduce antibiotic prescriptions if no antibiotics are prescribed in case of a positive viral test. Earlier investigations in adult populations have indicated that rapid antigen detection tests may contribute to a decrease in antibiotic prescriptions among adults evaluated in the ED [93]. Additionally, preliminary studies involving rapid influenza [94,95] or respiratory panel tests [96] have hinted at the potential for similar outcomes in children. However, better-designed recent pediatric trials failed to prove a reducing effect of rapid antigen detection testing on antibiotic prescriptions in children. Mattila et al. conducted a randomized study wherein children were allocated to either undergo viral testing (comprising 18 respiratory viruses and three bacteria with results available within 70 min) or receive standard care. The primary outcome assessed was the proportion of children prescribed antibiotics [97]. The study findings revealed no significant differences in antibiotic prescriptions between the intervention group (266 children, 27.3%) and the control group (28.5%; risk ratio 0.96; 95% CI 0.79–1.16). Similarly, Rao et al. conducted a comparable randomized controlled trial in Colorado, reporting no statistically significant difference in antibiotic prescription rates when the tests were used in a pediatric ED setting (RR 1.1; 95% CI 0.9–1.4) [98]. Furthermore, Tan et al. conducted a subanalysis of the data from the MOFICHE study, an observational multicenter investigation that included routine data from febrile children aged 0–18 years attending 12 European pediatric EDs [99]. Within this cohort, rapid antigen detection testing was performed in 1061 children (8%) and omitted in 11,463 children. Once again, the utilization of rapid viral testing was not found to be associated with an increased likelihood of antibiotic prescription (adjusted odds ratio 0.9, 95% CI 0.8–1.1).

These data should be approached with caution, as they do not negate the potential usefulness of rapid viral testing. The rationale supporting their utility, not to prescribe antibiotics for viral infections, remains robust. Several other studies, in fact, have demonstrated that syndromic rapid tests for respiratory pathogens are associated with lower antibiotic prescriptions, testing, or early suspension of antibiotics in children with positive viral results, suggesting that their proper interpretation may support clinicians [100]. In fact, viral infections are widely recognized as the primary cause of RTI. Recent extensive studies have demonstrated limited or no benefit from routine antibiotic administration for children with lower RTI, and shorter regimens may suffice in many cases of bacterial pneumonia [101]. It is plausible that additional factors affect the decision to prescribe antibiotics, including nonclinical or psychological considerations [102]. For instance, Covino et al. conducted a large-scale study involving pediatric patients and found that the mere decision to order certain diagnostic tests was associated with a significantly higher likelihood of receiving antibiotics, regardless of the test results [103]. Taken together, these observations imply that a multifaceted approach, involving various investigations and additional tools, may be necessary to enhance the appropriateness of antibiotic prescriptions.

### 4.3. Guidelines

Numerous guidelines exist for the management of common pediatric infections, encompassing a wide array of reasons for antibiotic prescription. Nonetheless, the impact of guideline implementation in the ED on pediatric antibiotic prescriptions has been the subject of limited high-quality research. Aronson et al. retrospectively analyzed the impact of institutional clinical practice guidelines on antibiotic prescription in infants younger than 56 days of age in 33 hospitals in the USA [104]. Their findings indicated that the use of ceftriaxone at ED discharge exhibited notable variations contingent upon adherence to clinical practice guidelines. This suggests that the implementation of guidelines can indeed exert an influence on antibiotic prescriptions. Similarly, Ayanruoh’s investigation highlighted the efficacy of a local guideline permitting the use of rapid streptococcal tests in a pediatric ED [105]. Their study demonstrated a substantial reduction in antibiotic utilization (41.38% in the pre-phase versus 22.45% in the post-phase, *p* < 0.0001). This reduction holds significance, particularly since pharyngitis remains a common basis for empirical antibiotic use [106]. In Spain, a similar initiative, founded on the implementation of local upper RTI guidelines, was linked to a decrease in antibiotic prescriptions [107]. However, the impact appeared most pronounced shortly after implementation, suggesting that ongoing interventions may be necessary. Conversely, studies in Italy by Donà et al. documented a tangible reduction in antibiotic prescriptions and an enhancement of the wait-and-see approach for acute otitis media, pneumonia, and pharyngitis [108,109]. Breakell et al. explored the effects of introducing the NICE bronchiolitis guidelines in a second-line hospital in England, comparing the periods before and after implementation [110]. Their findings revealed a substantial 16% absolute reduction in antibiotic prescriptions (from 22% to 6% of patients). This reduction was corroborated by studies conducted in Spain [111] and Rome [112]. However, in the USA, the implementation of the AAP bronchiolitis guidelines was associated with reduced utilization of diagnostics and medications but not antibiotics [113]. This suggests that while guidelines may constitute a valuable component in the endeavor to enhance antibiotic prescription practices, they may not be the sole solution to the challenge.

### 4.4. Decision Support Tools

Decision support tools (DSTs) offer another avenue for assisting clinicians in optimizing antibiotic prescribing within the ED. However, it is imperative to carefully refine their design during the pre-implementation phase. These tools should ideally encompass software capable of integrating various information sources, such as access to previous cultures for individual patients beyond the organization’s scope. Additionally, they should acknowledge that the role of an ED clinician extends beyond the confines of the ED, necessitating feedback on outcomes from colleagues working in both outpatient and inpatient settings [114]. Regarding ED practices, van de Maat et al. conducted a systematic analysis of clinical prediction models for childhood pneumonia, assessing their quality. Their findings revealed three clinical prediction models that incorporated clinical and laboratory markers for childhood pneumonia and demonstrated reasonably good discrimination between bacterial and viral infections, with sensitivities ranging from 79% to 84% [115]. DSTs that incorporate not only laboratory parameters but also clinical and demographic information appear to perform better than those relying solely on laboratory scores [116]. This observation likely reflects the absence of perfect biomarkers capable of unequivocally distinguishing between bacterial and viral infections. More recently, the MOFICHE group conducted a prospective analysis of a multivariable clinical prediction model designed to identify IBI in 12 EDs in eight European countries [117]. The model incorporated clinical symptoms, CRP, neurological signs, nonblanching rash, and comorbidity. It exhibited a rule-out threshold of 0.1%, characterized by a sensitivity of 97% and a negative likelihood ratio of 0.1 (95% CI 0.0 to 0.2). The rule-in threshold was set at 2.0%, with a specificity of 94% and a positive likelihood ratio of 8.4 (95% CI 6.9 to 10.0). Notably, in Tanzania, an algorithm founded on a limited set of clinical signs and point-of-care tests (including malaria rapid diagnostic tests, hemoglobin, oximeter, CRP, PCT, and glucose) was associated with a substantial 49% reduction in the relative risk of clinical failure compared to routine care. This approach also significantly reduced antibiotic prescriptions from 94.9% to 11.5% (*p* < 0.001) [117].

### 4.5. Audit and Feedback

Conducting regular rounds with multidisciplinary teams represents a potent strategy for improving antibiotic prescribing practices in diverse healthcare settings. These rounds are designed to facilitate discussions about the rationale for antibiotic use, the selection of appropriate agents, and the optimization of antibiotic therapy in terms of dosage, timing, and duration. Although such programs are increasingly implemented in numerous academic medical centers, they are predominantly concentrated within inpatient units and hospitals equipped with established AMS initiatives. An exemplary case is the Children’s Hospital Colorado, where daily face-to-face AMS programs have yielded a remarkable 10% reduction in antimicrobial usage during their initial four years of implementation, resulting in annual savings exceeding USD 1 million. In this context, clinicians engage in discussions with pharmacists regarding each antibacterial, antifungal, and antiviral prescription. They conduct daily rounds and scrutinize a list of patients that have garnered their attention, facilitating the exchange of ideas and recommendations for adopting more conservative treatment options [118]. However, the implementation of audits and feedback mechanisms can present challenges and ideally necessitates dedicated resources. This comprehensive approach entails optimizing workflow processes, reviewing antimicrobial usage patterns, examining microbiology reports, tailoring interventions to suit the local context, and integrating principles of implementation science to enhance sustainability and the likelihood of successful adoption during post-analytical phases. Furthermore, diagnostic effectiveness may evolve over time, influenced by factors such as the learning curve associated with diagnostic utilization and the introduction of novel therapies that enhance the impact of diagnostic results on clinical outcomes [119]. Crucially, there are some reports of efforts aimed at optimizing prescriptions within EDs. Jain et al. analyzed the impact of physician feedback on practice patterns relative to peers in a sizable adult ED. Their study revealed a reduction in resource utilization, including antibiotics, for various common ED conditions, all without detrimental effects on ED efficiency or the quality of care provided [120]. Likewise, a Norwegian team implemented an approach that combined audit and feedback with the distribution of pocket-sized guideline recommendations. This initiative led to a substantial increase in the appropriateness of empirical antibiotic prescriptions [121].

### 4.6. Follow-up Systems (Safety Netting)

One potential contributing factor to antibiotic overprescription in PEM is the perception that children, as a vulnerable demographic, can experience rapid deterioration or improvement in their condition. Moreover, infections frequently prompt revisits to the ED [122]. Consequently, owing to the uncertainty surrounding post-discharge outcomes and the nonspecific nature of early illness presentations, prescribing antibiotics may offer a sense of reassurance, including from a legal defensive standpoint. To address this issue effectively, the implementation of a systematic approach is imperative. Such an approach should provide clear guidance and information regarding post-discharge care instructions, when and how to seek further medical assistance, and should instill confidence in the discharging physician that the child is being discharged within a comprehensive safety net framework. This safety net system, extensively elaborated elsewhere [123,124,125], plays a pivotal role in enhancing the appropriateness of antibiotic prescriptions. Notably, the effectiveness of this approach has been extensively evaluated in the context of acute otitis media [126]. Adopting a wait-and-see approach coupled with a safety net, involving three days of observation before considering antibiotic treatment, has resulted in a high cure rate without antibiotics and significant caregiver satisfaction. Regrettably, similar experiences have not yet been explored for other prevalent pediatric infections, including lower RTI and pneumonia. Nevertheless, recent evidence from two trials conducted in low-to-moderate-income countries has shown limited benefit from routine antibiotic administration for children with pneumonia [127,128]. This suggests that a wait-and-see approach, coupled with a well-structured safety net, could theoretically be investigated for conditions beyond acute otitis media.

### 4.7. Training/Supervision

Certainly, at the core of AMS in PEM lies the imperative of continuous education regarding the indications for antibiotic prescription, clinical guidelines, and emerging tools for enhancing the customization of clinical management, including antibiotic prescription. For instance, a study by Frost et al. revealed that pediatricians consistently exhibit a higher propensity to employ antibiotics judiciously for the treatment of acute RTI compared to their counterparts in other medical specialties [129]. Additionally, a systematic review encompassing five studies found that pediatric emergency physicians consistently recorded significantly lower antibiotic prescription rates when contrasted with their general emergency medicine peers [130,131,132,133]. In contrast, a study by Covino et al., which examined a cohort of 51,633 pediatric patients discharged from the ED, uncovered an intriguing association [103]. It was discerned that patients assessed by physicians with over three years of pediatric expertise were more likely to receive antibiotic prescriptions, as indicated by an odds ratio of 1.22 (95% CI 1.13–1.31, *p* < 0.001), compared to their less experienced colleagues. Similar findings were found in the USA, where antibiotics were more commonly prescribed by staff physicians than trainees and more frequently in nonteaching than teaching hospitals [134].

## 5. Evaluation of Antibiotic Consumption and AMS Techniques in PEM

Accurate measurement of antibiotic usage is indispensable for comprehending prescription patterns, evaluating AMS interventions, and establishing meaningful benchmarks [135]. Yet, measuring antibiotic use in PEM poses several challenges. Subsequent to ED attendance, patients may undergo further care either as inpatients or in the outpatient setting. While electronic health records provide abundant data for evaluating prescription practices in inpatient settings, such as medication administration charts, the assessment of prescription practices in PEM encounters difficulties due to limited data sources and resource constraints. Furthermore, there is presently no singular universally accepted metric serving as a comprehensive measure for antibiotic prescription to children in ED settings [136].

In a broad context, currently utilized metrics (Table 2) aim to capture either the quantity or quality of antibiotic prescriptions. The defined daily dose (DDD) stands as the World Health Organization’s (WHO) preferred metric for evaluating inpatient antibiotic consumption [137]. However, as this metric is grounded in a fixed adult dose, it does not apply to the pediatric population. Furthermore, its utility diminishes when different doses are utilized for varying indications. Alternative metrics, such as days of therapy (DOT) and the length of antibiotic course, address some of the limitations of DDD. Nevertheless, standardization for factors like population size or prescriber characteristics is required to account for inherent differences across populations [138]. For instance, antibiotic usage will inevitably differ between a tertiary care hospital and a community hospital, and medical prescribers may prescribe different types of antibiotics than surgical specialists. A limitation of using both DDD and DOT is that it incentivizes the use of broad-spectrum antibiotics rather than combinations of small-spectrum antibiotics with similar coverage.

In the context of outpatient care, quantitative metrics include the total number of antibiotics prescribed per patient population, but this may inadequately reflect prescribing practices, as it heavily depends on the healthcare setting [136]. For example, it underestimates antibiotic use in settings where patients are seen with lower incidences of infections, such as surgical patients or well infants. Additionally, reliance on prescription data can overestimate utilization rates in outpatient settings, as patients may not fill their prescriptions.

Moreover, quantitative metrics lack detailed information on prescription indications, limiting their effectiveness in setting improvement targets. Therefore, more specific quantitative metrics, such as the percentage of visitors for RTI receiving prescribed antibiotics, can be employed. However, diagnostic selection bias may arise when prescribers adapt the registered working diagnosis to justify an antibiotic prescription. For instance, an infant clinically diagnosed with bronchiolitis may be registered as pneumonia if the prescriber aims to justify an antibiotic prescription.

Alternatively, the index of broad-spectrum to narrow-spectrum antibiotics is useful in settings favoring narrow-spectrum antibiotics. More recently, the amoxicillin index was proposed as a metric to evaluate the appropriateness of antibiotic prescription in children, as amoxicillin is the first-line antibiotic for various common infections in childhood, such as acute otitis media and pneumonia [135]. However, this metric does not adequately express the appropriateness of prescribing in other infections, such as UTIs or skin and soft tissue infections, where other antibiotics are first-line agents. Nevertheless, all quantitative metrics lack a detailed insight into the reasons for antibiotic prescription. On the contrary, qualitative metrics provide more insight into these reasons but require more detailed information on prescriptions and therefore more resources. Furthermore, these metrics do require a standard or guideline to assess prescriptions. Examples of qualitative metrics include the percentage of prescriptions concordant with guidelines, the proportion of prescriptions that are not indicated, or the percentage of prescriptions in agreement with microbiological findings.

Brigadoi et al. conducted a systematic review to assess the efficacy of AMS programs in PEM and primary care settings [139]. The review encompassed 59 studies, with 15 focusing on AMS program implementation in the ED and the remaining studies in primary care settings. The predominant AMS interventions employed across the studies included guideline implementation, education, and audits with feedback. The most commonly reported outcome metrics were antibiotic prescription rates, compliance with guidelines, and changes in healthcare expenditures. Across the majority of studies, improvements in these outcomes were observed during the study periods. Ensuring the sustained impact of AMS programs necessitates ongoing analysis of outcome metrics.

**Table 2:** Currently available metrics for antibiotic use.

Metric	Definition
Quantitative	
Defined daily dose (DDD)	The assumed average maintenance dose per day for a drug for its main indication in adults [137].
Defined daily dose per defined population	DDD standardized for population size (e.g., per 100 bed days) [137].
Days of therapy (DOT)	The number of days that a patient receives an antibiotic course, regardless of the dose. If a patient is prescribed multiple antibiotics, each antibiotic counts as a DOT [138].
DOT per defined population	DOT standardized for population size [138].
Proportion of patients exposed to antibiotics	Number of patients exposed to at least one antibiotic proportional to the total number of patients [138].
Proportion of visit with an antibiotic prescribed	Percentage of outpatient contacts in which a patient receives at least one antibiotic prescription [135].
Proportion of acute RTI visits prescribed antibiotics	Percentage of outpatient contacts for RTI in which a patient receives at least one antibiotic prescription [135].
Prescriptions per defined population	Number of prescriptions, independent of the prescribed dose, per defined population [140].
Prescriptions per defined number of physician contacts	Number of prescriptions, independent of the prescribed dose, standardized for the number of physician contacts [135].
Treatments/courses per defined population or per defined number of physician contacts	Number of treatment courses (filled prescriptions), independent of prescribed dose, per defined population or per defined number of physician contacts [140].
Broad/narrow ratio	Proportion of patients receiving broad-spectrum antibiotics compared to those receiving narrow-spectrum antibiotics [135].
Amoxicillin index	Number of amoxicillin prescriptions divided by all antibiotic prescriptions [135].
Qualitative	
Appropriateness of antibiotic prescriptions	Percentage of antibiotic prescriptions that are assessed appropriate [136].
Adherence to guidelines	Percentage of antibiotic prescriptions adherent to guidelines [135].

## 6. Conclusions

Antibiotic overprescribing remains a common practice in PEM, fueled by pressures on EDs worldwide, the perceived pressure of patients and caregivers, diagnostic uncertainty, and decision fatigue in prescribers. As the use of antibiotics invariably contributes to adverse drug effects, increased healthcare expenditures, and the emergence of antimicrobial resistance, AMS interventions targeting antibiotic prescriptions become imperative. While these interventions have been incorporated into inpatient healthcare settings, they encounter practical challenges in PEM.

In this review, we explored the diagnostic decision process in PEM and delved into potential AMS techniques, such as biomarkers, host RNA signatures, rapid microbiology tests, decision support tools, guideline implementations, audit and feedback, and safety netting systems. Recognizing the need for advancements, integrating artificial intelligence and machine learning methodologies into AMS interventions holds promise for optimizing antibiotic prescription practices in PEM. These techniques can assist in real-time decision support and identifying patterns in antibiotic use [141,142].

Evaluating antibiotic use in PEM settings is crucial to define targets for stewardship but faces unique challenges, such as limited data sources and resource constraints. Further research on antibiotic use in PEM settings will define appropriate metrics for antibiotic consumption in this context.

## Data Availability

Not applicable.
